# Low percentage of clinically relevant pistachio nut and mango co-sensitisation in cashew nut sensitised children

**DOI:** 10.1186/s13601-017-0145-z

**Published:** 2017-03-20

**Authors:** J. P. M. van der Valk, R. el Bouche, R. Gerth van Wijk, H. de Groot, H. J. Wichers, A. E. J. Dubois, N. W. de Jong

**Affiliations:** 1 Department of Internal Medicine, Section of Allergology, Erasmus MC, Office Gk 323, P.O. Box 2040, 3000 CA Rotterdam, The Netherlands; 2 Department of Paediatric Allergology, Diaconessenhuis Voorburg, RdGG, Delft, The Netherlands; 3 Food and Biobased Research, Wageningen University and Research Centre, Wageningen, The Netherlands; 4 Department of Paediatric Pulmonology and Paediatric Allergology, University Medical Centre Groningen, GRIAC Research Institute, University of Groningen, Groningen, The Netherlands

**Keywords:** Cashew nut allergy, Children, Co-sensitisation, Food challenge test, Mango allergy, Pistachio nut allergy

## Abstract

**Background:**

Cashew nut, pistachio nut and mango belong to the *Anacardiaceae* family and are botanically related. Therefore, cashew nut sensitised children are frequently advised to eliminate cashew nuts and pistachio nuts from their diet. The ‘Improvement of Diagnostic mEthods for ALlergy assessment (IDEAL trial number NTR3572) study showed that cashew nut sensitised children were co-sensitised to pistachio nut in 98% of cases and to mango in 21% of cases. The aim of this follow-up study to IDEAL is to assess the clinical relevance of co-sensitisation to pistachio nut and mango in cashew nut sensitised children.

**Methods:**

Children were recruited from the study: ‘Improvement of Diagnostic mEthods for ALlergy assessment (IDEAL trial number NTR3572). Inclusion criterion for the IDEAL study was sensitization to cashew nut as demonstrated by either SPT or sIgE, and a clinical history of reactions to cashew nuts or no previous (known) exposure. Sensitized children who were tolerant to cashew nuts were excluded. Inclusion criterion for this IDEAL follow-up study was co-sensitization to pistachio nut, regardless the result of the DBPCFC with cashew nut. In this follow-up study a double-blind placebo-controlled food challenge with pistachio nut and an open food challenge with mango were performed.

**Results:**

Twenty-nine children (mean age of 11.6 years, 62% male) were included. Pistachio nut sensitisation was clinically relevant in only 34% of cashew-sensitised children and only 31% of cashew challenge positive children. None of the children was challenge positive to mango.

**Conclusion:**

Although co-sensitisation between cashew nut and pistachio nut was observed in 98%, pistachio nut sensitisation was only clinically relevant in 34% of the children. Therefore, a challenge test with pistachio nut is recommended in children with cashew nut and pistachio nut sensitisation.

*Trial registration* The study was registered in the Dutch trial register (registration number 3572) on 10 August 2012 (retrospectively registered)

## Background

 Cashew nut, pistachio nut and mango belong to the *Anacardiaceae* family and are botanically related. Cashew nut allergic children are frequently advised to eliminate not only cashew nuts, but also pistachio nuts from their diet. This advice is based on the sensitisation pattern and possible cross-reactivity [1, 2]. Cross-sensitisation between cashew nut and pistachio nut has been previously established by specific IgE (sIgE) inhibition tests [3–6]. However, studies that confirm clinical cross-reactivity by performing a challenge are rare [7].

The aim of this follow-up study of the ‘Improvement of Diagnostic mEthods for ALlergy assessment (IDEAL) study’ (trial number NTR3572) is to assess the clinical relevance of pistachio nut- and mango sensitisation in cashew nut sensitised children. The IDEAL-study showed that children sensitised to cashew nut were co-sensitised to pistachio nut in 98% (169/173) and to mango in 21% (37/173).

## Methods

### Patient selection and study design

Children were recruited from the multi-centre IDEAL-study and were included between January 2015 and June 2015. Inclusion criterion for the IDEAL study was sensitization to cashew nut as demonstrated by either SPT or sIgE, and a clinical history of reactions to cashew nuts or no previous (known) exposure. Sensitized children who were tolerant to cashew nuts were excluded. Inclusion criterion for this IDEAL follow-up study was co-sensitization to pistachio nut, regardless the result of the double-blind placebo-controlled food challenge (DBPCFC) with cashew nut [8]. For practical reasons only two of the three centres were invited to participate in this pistachio nut and mango follow-up study. Medical history, sensitisation results (sIgE and SPT), and results from the DBPCFC with cashew nut were obtained from the IDEAL-study. The children underwent a DBPCFC with pistachio nut and an open food challenge with mango in this follow-up study. Prior to the pistachio and mango challenge a dietary history questionnaire was used on consumption of pistachio and mango [9] Medical ethical approval was obtained in January 2015. Parents of children (2–12 years old) and parents and children (≥12 years old) signed the informed consent.

### Skin prick test

All children underwent a SPT in the IDEAL study with cashew nut and pistachio nut extract and mango juice, a positive control (histamine 10 mg/ml ALK-Abello, Nieuwegein, the Netherlands) in duplicate and a negative control. Cashew nuts (roasted, unsalted) and pistachio nuts (fresh, not roasted, unsalted nuts) were homogenized mechanically, ground with a mortar and pestle, defatted by ether extraction, and subsequently air-dried. A 10% w/v extract in phosphate-buffered saline with the pre-treated material was made. Mango juice was prepared from pieces of ripe mango fruit pulp, without skin or kernel [8]. The SPT was performed by applying a drop of the allergen extract on the skin of the volar aspect of the forearm; subsequently the epidermis was punctured with a standardised 1 mm sharp tip sterile lancet.

We used a precise scanning method to ascertain the SPT results. We divided the area of the allergen-induced wheal by the mean area of two positive histamine-induced wheals. This ratio is defined as HEP-index area. A HEP-index area of ≥0.4 corresponding with a wheal diameter of ≥3 mm was considered positive [10].

### Specific IgE

In the IDEAL study serum samples were analysed for sIgE to cashew nut, pistachio nut and mango using the Siemens IMMULITE 2000 XPi Immunoassay System (Med. Imm. Laboratory; Reinier de Graaf Groep (RdGG). Levels above 0.35 kU/l were considered positive.

### Challenge tests with cashew nut in the IDEAL study and pistachio nut in the follow-up study

All children underwent a DBPCFC with cashew nut (in the IDEAL study) and pistachio nut (follow-up study). The food challenge consisted of an eight-step incremental dose regime. The time interval between each step was 30 min. The challenge recipe and dosages used for the DBPCFC with pistachio nut were based on validated and standardised cashew nut recipes used in the IDEAL study, and contained the same amount of protein equivalent [11]. Roasted unsalted cashew nuts and pistachio nuts were provided by Intersnack, Doetinchem, the Netherlands. The food matrix (muffin dough) mainly consisted of wheat, sugar, gingerbread spice mix and coconut. The challenge dose schedule of the pistachio is shown in Table 1. Children under the age of 4 years were not required to complete step 8.

**Table 1:** Challenge doses DBPCFC with pistachio nut and mango

	Pistachio protein (mg)	Pistachio protein cum (mg)	Average amount of Pistachio nut	Mango protein (mg)	Mango protein cum (mg)	Mango volume (gr)
Dose 1	1	1	0.007^a^	3	3	0.3^b^
Dose 2	3	4	0.02	10	13	1
Dose 3	10	14	0.07	30	43	3
Dose 4	30	44	0.20	100	143	10
Dose 5	100	144	0.67	300	443	30
Dose 6	300	444	2.08	1000	1443	100
Dose 7	1000	1444	6.94	–	–	–
Dose 8	1736	3180	12.06	–	–	–

^a^The average weight of one pistachio nut was approximately 700 mg, with 23.8 g pistachio protein per 100 g pistachio nuts (Intersnack the Netherlands B.V.)

^b^100 g mango (with kernel and peel) contains approximately 1.0 g mango protein

### Challenge test with mango

Children with no history of symptoms after the consumption of mango were considered non-allergic. The remaining children underwent an open challenge (OFC) with mango regardless sensitization to mango. The food challenge with mango (pieces of fruit without skin or kernel) consisted of a six-step incremental dose regime. The challenge dose schedule is shown in Table 1.

### Statistical analysis

Statistical analyses were done with the Fisher exact test, Mann–Whitney U test and Chi square test. All analyses were performed with IBM SPSS Statistics, version 21.

## Results

### Patient selection

Eighty-five children sensitised to cashew nut and pistachio nut, from two of the three participating centres of the IDEAL-study were asked to participate in the pistachio nut and mango follow-up study. The average period between participation in the IDEAL study and this follow up study was 24 months. A total of 29 children (34%) participated, 38 (45%) did not respond to the invitation, 16 (19%) refused to participate citing reasons such as that the food challenge was time-consuming and burdensome for the child or there was fear for a reaction during the challenge. Two children were ultimately excluded because they did not receive all interventions.

### Patient characteristics and diagnostic results from the IDEAL study

Twenty-nine children, 18 boys (62%), mean age of 11.6 years (range 4–20 years) were included. Symptoms, consistent with eczema, asthma or hay fever were reported by 15/29 (52%), 7/29 (24%) and 14/29 (48%) of the children, respectively. None of the children in this group consumed pistachio nuts.

The median cashew nut sIgE was 5.28 kU/l (range 0.4–100 kU/l) and the median pistachio nut sIgE was 7.25 kU/l (range 0.6–82 kU/l). The median SPT HEP-index area of cashew nut and pistachio nut was 2.50 (range 0–8.8) and 2.02 (range 0–9.4), respectively. Twelve of the 29 children were co-sensitised to mango with a median sIgE to mango of 0.71 kU/l (range 0–3.76) and a median SPT HEP-index area of 0.46 (range 0–1.45).

In order to exclude selection bias, we compared the patient characteristics and diagnostic results of the participating children (N = 29) with the non-participating children. There was no significant difference in gender (p = 0.80), age (p = 0.08), asthma (p = 0.10), eczema (p = 0.52), hay fever (p = 0.57), sIgE to cashew nut (p = 0.85), sIgE to pistachio nut (p = 0.71), SPT cashew nut (p = 0.50) and SPT pistachio nut (p = 0.13).

### Food challenge with cashew nut (IDEAL study) versus pistachio nut (follow-up study)

The pistachio nut DBPCFC (follow-up study) was positive in 10/29 (34%) and negative in 19/29 (66%) children (Table 2). Most children with a positive DBPCFC with pistachio nut experienced ‘oral allergy’ symptoms followed by gastro-intestinal symptoms, skin symptoms and upper airway symptoms. Eight of ten children experienced both objective and subjective symptoms and an anaphylactic reaction according to the EAACI Guidelines for Anaphylaxis [12] occurred in one child.

**Table 2:** Outcome of the DBPCFC with cashew nut, pistachio nut and OFC with/or home consumption of mango

	Outcome DBPCFC pistachio nut			Outcome OFC mango^a^	
	Positive	Negative	Total	Positive	Negative
Outcome DBPCFC cashew					
Positive	9/29 (31%)	13/29 (45%)	22/29 (76%)	0	10/11 (91%)
Negative	1/29 (3%)	6/29 (21%)	7/29 (24%)	0	1/11 (9%)
Total	10/29 (34%)	19/29 (66%)	29 (100%)		11/29 (38%)

^a^17 children already consumed mango without problems, therefore a challenge was not indicated, 1 child declined to undergo the OFC

Of the 29 children, 22 children (76%) had a positive challenge with cashew nut (in the IDEAL study) and 7 children (24%) had a negative challenge with cashew nut. Only 9 of the 29 children (31%) had positive challenge with cashew nut as well as a positive challenge with pistachio nut (Table 2). In 6 of the 22 (27%) children, an anaphylactic reaction occurred during the cashew nut challenge. These 6 children had in all cases a negative pistachio nut food challenge outcome. None of these results were statistically significant.

Since the time interval between the cashew nut challenge and the pistachio nut challenge was rather long (median 24 months), it may be argued that the tolerance we observed to pistachio nut was due to spontaneous tolerance due simply to the passage of time. We thus analyzed whether the time between the DFBPCFCs was related to tolerance and whether greater proportions of negative DBPCFCs were seen with increasing time intervals between the challenges. There was no significant correlation between the time between the DBPCFCs and the proportion of negative test results to pistachio (Spearman correlation coefficient = 0.07, p = 0.7).

### Food challenge with mango

Seventeen children already consumed mango without problems, therefore a challenge was not indicated. In this group 7 (41%) children were sensitized. One declined to undergo the OFC because it was time-consuming. In total, 11 children participated in the OFC with mango and in all cases the challenge was negative. Of this group only 4 children (36%) were sensitized.

### Food challenge results versus sIgE levels

In patients with a positive challenge to both, cashew nut (IDEAL study) and pistachio nut (Follow-up study) (n = 9), the median sIgE amounted 10.90 and 17.10 kU/l, respectively. Significantly lower median sIgE values were found in children reacting to either cashew nut (2.48 kU/l or pistachio nut (3.77 kU/l), with p values of 0.014 and 0.024, respectively.

## Discussion

To our knowledge, this is the first study using DBPCFCs with pistachio nuts in children to ascertain the clinical relevance of co-sensitization to pistachio nut in cashew nut sensitized children.

We demonstrated that only one third of the children with this co-sensitization to pistachio nuts has a challenge proven pistachio nut allergy.

The time period of median 24 months between both studies is rather long, but the low number of reactors to pistachio is not likely to be caused by the development of spontaneous tolerance over time, as there was no significant association between the time period between the challenges and the outcome.

Previous studies reported high percentages of co-sensitisation and cross-sensitisation between cashew nut and pistachio nut [1, 3–6, 13–15], however the studies were only performed in very small groups, and none of the studies performed cashew nut and pistachio nut challenges. Two case studies on patients with cashew nut-pistachio nut allergy, investigated cross reactivity between the major allergens Ana o 1 from cashew nut and Pis v 3 (7S vicilin-like protein) [4, 5]. Willison et al. performed inhibition dot- and Western immunoblot assays, and found complete cross-reactivity between rAna o 1 and rPis v 3 in the serum of cashew/pistachio allergic patients.

To further investigate the cross-reactivity between cashew nut and pistachio immunoblot inhibition studies are necessary. Although the results in this study suggest that cashew nut is the primary sensitizer, we have found one patient with a positive pistachio challenge and a negative cashew challenge, raising possibility that pistachio may occasionally be the primary sensitizer. Children with an allergy to cashew nut and co-sensitisation to pistachio nut are often advised to eliminate both nuts from the diet. Based on our results this might not be the best approach. Although higher levels of (co-) sensitisation to cashew nut and pistachio nut are associated with a higher risk of clinical allergy to these nuts, a DBPCFC is clearly indispensable for making a definitive diagnosis in individual patients.

Apart from the need for a well-established diagnosis, unnecessary avoidance of pistachio nuts or mango may have other disadvantages. Unnecessary lengthy elimination of allergenic foods in sensitised patients can increase the risk of a severe allergic reaction after accidental intake of the allergen [15].

In conclusion, this is the first multi-centre study in patients sensitised to cashew nut investigating the clinical relevance of co-sensitisation to pistachio nut and mango with food challenges. The percentage of clinical relevant co-sensitisation was low (34%) for pistachio nut and absent for mango in this study population. Oral food challenges are recommended in order to avoid unnecessary avoidance of pistachio nut in cashew nut allergic patients.

## Abbreviations

DBPCFC: double-blind placebo-controlled food challenge
HEP: histamine equivalent prick
IDEAL: Improvement of Diagnostic mEthods for ALlergy assessment
OFC: open food challenge
sIgE: specific IgE
SPT: skin prick test
